# Bench to bedside review of myositis autoantibodies

**DOI:** 10.1186/s12948-018-0084-9

**Published:** 2018-03-07

**Authors:** Boaz Palterer, Gianfranco Vitiello, Alessia Carraresi, Maria Grazia Giudizi, Daniele Cammelli, Paola Parronchi

**Affiliations:** Experimental and Clinical Medicine Department, University of Florence, Largo Brambilla 3, 50134 Florence, Italy

**Keywords:** Myositis, Autoantibodies, Immunofluorescence, Anti-nuclear antibodies, Dermatomyositis, Polymyositis, Immune-mediated necrotizing myopathy, Inclusion body myositis, MDA5, HMGCR

## Abstract

Idiopathic inflammatory myopathies represent a heterogeneous group of autoimmune diseases with systemic involvement. Even though numerous specific autoantibodies have been recognized, they have not been included, with the only exception of anti-Jo-1, into the 2017 Classification Criteria, thus perpetuating a clinical-serologic gap. The lack of homogeneous grouping based on the antibody profile deeply impacts the diagnostic approach, therapeutic choices and prognostic stratification of these patients. This review is intended to highlight the comprehensive scenario regarding myositis-related autoantibodies, from the molecular characterization and biological significance to target antigens, from the detection tools, with a special focus on immunofluorescence patterns on HEp-2 cells, to their relative prevalence and ethnic diversity, from the clinical presentation to prognosis. If, on the one hand, a notable body of literature is present, on the other data are fragmented, retrospectively based and collected from small case series, so that they do not sufficiently support the decision-making process (i.e. therapeutic approach) into the clinics.

## Background

The detection of autoantibodies in autoimmune diseases, either systemic or organ specific, can have both diagnostic and prognostic importance. Some autoantibodies have a clear pathogenic role, such as anti-erythrocyte membrane proteins antibodies in autoimmune hemolytic anemia and anti-dsDNA in Systemic Lupus Erythematosus (SLE). However, the presence of autoantibodies is more frequently considered to be an epiphenomenon, even though their detection plays a critical role for the diagnosis of some connective tissue diseases (CTDs), [i.e. anti-SSA/Ro in Sjögren syndrome (SjS) and anti-Sm in SLE], as included into the classification criteria of CTDs [1, 2]. Repeated serum sampling might be informative about the clinical course of the disease and response to immunosuppressive therapy, as in the case of anti-dsDNA antibodies in SLE. Furthermore, the presence of some autoantibodies could help to discriminate specific clinical patterns within the same disease, as with diffuse and limited cutaneous systemic sclerosis (SSc) and their relationship with anti-Scl-70 and anti-centromere, respectively.

In idiopathic inflammatory myopathies (IIMs), albeit anti-Jo-1 autoantibody was discovered more than thirty years ago, the percentage of patients in whom an autoantibody could not be recognized (the so called “serologic gap”) was still high until recently. IIMs have been historically divided in polymyositis (PM) and dermatomyositis (DM) on a purely clinical basis despite phenotypic variability.

Due to this heterogeneity, the numerous autoantibodies and unavailability of reliable assays in all the laboratories, the clinical use of serology lagged behind and autoantibodies are not part of the most recent IIM Classification Criteria [3].

A comprehensive review regarding the clinical features, diagnostic work-up and relationship to some peculiar autoantibodies has been recently published by Milone [4].

## Myositis-specific and –associated autoantibodies: definitions

Autoantibodies found in IIM patients have been classified into two main categories: myositis-specific autoantibodies (MSAs), which can be found in IIMs exclusively, and myositis-associated autoantibodies (MAAs), which can also be found in other CTDs [5, 6]. MSAs and MAAs are summarized in Table 1.

**Table 1:** Summary of the main features of MSAs and MAAs

Antibody	Antigen	IP		IIF	Clinical association
		Proteins (kDa)	RNA	HEp-2	
Myositis-specific autoantibodies (MSAs)					
Anti-Jo-1	Histidyl-tRNA synthetase	50	tRNA^His^	Cytoplasmic fine speckled	Classic anti-synthetase syndrome with more frequent muscle involvement
Anti-PL-7	Threonyl-tRNA synthetase	80	tRNA^Thr^	Cytoplasmic dense fine speckled	Anti-synthetase syndrome with prevalent ILD
Anti-PL-12	Alanyl-tRNA synthetase	110	tRNA^Ala^	Cytoplasmic dense fine speckled	Anti-synthetase syndrome with prevalent ILD
Anti-EJ	Glycyl-tRNA synthetase	75	tRNA^Gly^	Cytoplasmic speckled	Anti-synthetase syndrome
Anti-OJ	Isoleucyl-tRNA synthetase	150 + 170/130/75	tRNA^Iso^	Cytoplasmic speckled	ILD alone or anti-synthetase syndrome
Anti-KS	Asparaginyl-tRNA synthetase	65	tRNA^Asp^	Cytoplasmic speckled	ILD alone or anti-synthetase syndrome
Anti-Zo	Phenylalanyl-tRNA synthetase	60/70	tRNA^Phe^	Cytoplasmic speckled	Myositis
Anti-YRS/HA	Tyrosyl-tRNA synthetase	59	tRNA^Tyr^	Cytoplasmic speckled	Myositis
Anti-Mi-2	Nucleosome Remodelling Deacetylase (NuRD) (Mi-2α/β)	240 + 200/150/75/65/63/50/34		Fine speckled	Classical DM
Anti-SAE	Small ubiquitin-like modifier activating enzyme (SAE1/2)	40/90		Fine speckled	Severe cutaneous disease that classically precede DM with severe dysphagia and systemic symptoms
Anti-MDA5 (anti-CADM140)	Melanoma Differentiation-Associated gene 5 (MDA5)	140		Negative or Cytoplasmic speckled	Hypo-amyopathic, ILD with possible RP-ILD and severe and peculiar skin involvement
Anti-TIF1γ/α (anti-p155/p140)	Transcription intermediary factor 1 (TIF1γ/α)	155/140		Fine speckled	Juvenile DM. Cancer-associated hypo-myopathic DM
Anti-TIF1β	Transcription intermediary factor 1β	120		Fine speckled	DM
Anti-NXP2 (anti-MJ)	Nuclear matrix protein (NXP-2)	140		Fine speckled and/or multiple nuclear dots	Juvenile DM, diffused calcinosis. Cancer-associated DM
Anti-SRP	Signal recognition particle	72/68/54/19/14/9	7SL	Cytoplasmic dense fine speckled	IMNM with frequent esophageal involvement. Possible ILD
Anti-HMGCR	HMG-CoA reductase	200/100		Negative or Cytoplasmic speckled	IMNM with or without history of statin exposure
Myositis-associated autoantibodies(MAAs)					
Anti-PM-Scl	Exosome protein complex (PM/Scl75/100)	75/100		Nucleolar homogeneous	Overlap PM/SSc
Anti-C1D	Exosome associated protein				Overlap PM/SSc
Anti-U1-RNP	U1 small nuclear RNP	11–70	U1	Coarse speckled	MCTD
Anti-fibrillarin (anti-U3-snRNP)	Fibrillarin	34	U3	Nucleolar clumpy	SSc
Anti-Ku	DNA-PK regulatory subunit	70/80		Fine speckled	PM/SSc. Potentially severe ILD
Anti-Ro52	Ro-52/TRIM21	52		Negative, fine speckled or cytoplasmic speckled	ILD. Frequently coupled with other MSA
Anti-Ro60/SSA	Ro-60/SS-A	60		Fine speckled	SjS, SLE
Anti-La/SSB	SS-B	48		Fine speckled	SjS, SLE
Anti-cN-1A (anti-Mup44)	Cytosolic 5′nucleotidase 1A				sIBM
Miscellaneous					
Anti-RuvBL1/2	RuvBL1/2 complex	48/49		Speckled	SSc, PM, Morphea
Anti-Su/Ago2	Argonaute 2	100/102 and 200		Cytoplasmic discrete dots	ILD in absence of cancer. Frequently coupled with MSA, Ro-52 and other antibodies
Anti-SMN	Survival of Motor Neuron	38 + 130/120/33		Few nuclear dots	PM/SSc
Anti-NUP	Nup358/RanBP2, gp210, Nup90, p200/p130, Nup62			Punctate nuclear envelope	Subgroup of PM/SSc patients (so called NUP-syndrome). PBC
Anti-mitochondrial (AMA-M2)	Branched-chain α-ketoacid dehydrogenase complex			Cytoplasmic reticular/AMA	Long-lasting myositis with muscle atrophy and cardiac involvement. PBC
Anti-KJ	Translocation factor	30/43		Cytoplasmic speckled	Anti-synthetase-like syndrome
Anti-Fer (anti-eEF1)	Eukaryotic elongation factor 1				Anti-synthetase-like syndrome
Anti-Wa		48		Cytoplasmic speckled	Anti-synthetase-like syndrome
Anti-Mas	selenocysteine seryl-tRNA-protein complex	48	tRNA^[Ser]Sec^	Cytoplasmic speckled	Non-immune mediated rhabdomyolysis. Autoimmune hepatitis
Anti-PMS	DNA repair mismatch enzyme (PMS1, PMS2, MLH1)				Mild myositis
Anti-cortactin	Cortactin	68			PM. Myasthenia gravis
Anti-FHL1	Four-and-a-Half LIM domain 1				Myositis and muscular atrophy with severe systemic involvement

There is no agreement about the attribution of rare and newly discovered autoantibodies to either MSAs or MAAs group [7]. Anti-synthetase autoantibodies (ARS) themselves, especially anti-PL-7, PL-12 and KS, often detected in interstitial pneumonia with autoimmune features (IPAF) patients, independently from muscular involvement, are still discussed as MSAs [8].

The MAAs group contains Anti-Pm-Scl, U1/U2RNP and Ku, which are associated with overlap syndromes with muscular involvement [9]. Anti-fibrillarin and anti-U1-snRNP are sometimes considered as MAAs, even though they are more specific for the diagnosis of SSc and mixed connective tissue disease (MCTD), respectively [10, 11]. Anti-Ro52 are usually considered a MAA, even though they are more frequently found in association with other MSA (ARS, anti-MDA5 and anti-SRP, in particular) [12], and define a peculiar clinical spectrum in which the lung involvement is more common than the muscular one [13].

## Detection methods

There are several methods to test for MSAs and MAAs, with variable sensibility, specificity, costs, complexity and feasibility in clinical and research settings. Indirect immuno-fluorescence (IIF) on HEp-2 cells, counter-immuno-electrophoresis (CIE), immuno-diffusion (ID) and immuno-enzymatic assays such as enzyme-linked immunosorbent assay (ELISA), fluorescent enzyme-linked assay (FEIA) and chemiluminescent immuno-assay (CLIA) are the most commonly adopted systems in diagnostic laboratories. However, immuno-precipitation (IP) of RNAs with silver staining and/or protein IP of cellular lysates (usually K562 cells) radiolabeled with ^35^S-methionin, is the gold standard for most antibodies. In order to streamline the detection of many autoantibodies at the same time in a cost/effective manner, recent multiplex assays, like immunoblots (IB) and Addressable Laser Beads Immuno Assay (ALBIA) have been developed [14].

Anti-nuclear autoantibodies (ANA) determination by IIF is virtually universally available and it can be considered an accessible screening method for many MSAs and MAAs [15]. Furthermore, the recognition of particular IIF patterns can hint to some specific autoantibodies. However, using ANA IIF as the sole screening method for MSAs/MAAs, is not recommended because of low sensitivity, very low specificity and/or lack of antigen expression by HEp-2 cells [16]. In addition to this, ANA IIF is burdened by reproducibility issues due to the operator-dependent recognition of rare patterns and variation among different commercial HEp-2 substrates [17].

CIE and ID have historically been the first methods to detect specific MSAs/MAAs. Even though they identify numerous specificities within a single assay, they are semi-quantitative, work-intensive and scarcely sensitive [14]. For all those reasons, they have been largely substituted by immunoenzymatic tests.

The main advantages of ELISAs are standardization, large-scale reproducibility and quantitative results. The disadvantage of the conjugation of antigens to a substrate resides on the possible loss of conformational epitopes and/or the formation of neo-epitopes, which may in turn impact the test performance [14].

Immunoblot (IB) assays can simultaneously test for many autoantibodies, albeit the denaturation of proteins during gel preparation implicates the recognition of linear epitopes only [14].

Commercial multiplex IBs, like dot blots or line blot assays (LIA), based on recombinant or synthetic peptides have been increasingly available, benefitting from pure antigens not requiring a gel passage [18, 19].

IP is the gold standard as it evaluates the binding of the autoantibodies to the RNA and protein complexes in their native conformation, yielding the best sensitivity and specificity [20]. The major limitations of IP are due to technical difficulties, costs and use of radioactive reagents [20]. In addition, interpretation may be complex because the target antigen can co-precipitate with non-target complexed proteins, with the consequence of multiple IP bands [20]. Thus, a comparison with reference sera or further purification and characterization with other methods (i.e. mass spectrometry) is necessary [14]. Quantitative PCR of reverse transcribed RNA components extracted from standard IP can be also used as a detection method for autoantibodies binding ribonucleoproteic complexes [21].

## Myositis-specific autoantibodies

### Anti-synthetases autoantibodies

ARS are a group of autoantibodies directed against the aminoacyl transfer RNA (tRNA) synthetases, which are amino acid-charging enzymes. Autoantibodies to eight tRNA synthetases have been discovered so far: histidyl (Jo-1), threonyl (PL-7), alanyl (PL-12), glycyl (EJ), isoleucyl (OJ), asparaginyl (KS), phenylalanyl (ZO), and tyrosyl (YRS/HA) tRNA synthetases [22].

Anti-Jo-1 antibodies were identified by double immune-diffusion (DID) of calf thymus extract in 1980 and were the first MSAs described [23]. IP represents the gold standard for their identification with the following protein bands: Jo-1 50 kDa, PL-7 80 kDa, PL-12 110 kDa, EJ 75 kDa, OJ 150 kDa and a multi-enzyme complex of 170, 130, and 75 kDa, KS 65 kDa, ZO 60/70 kDa, YRS/HA 59 kDa [22].

**Fig. 1:**
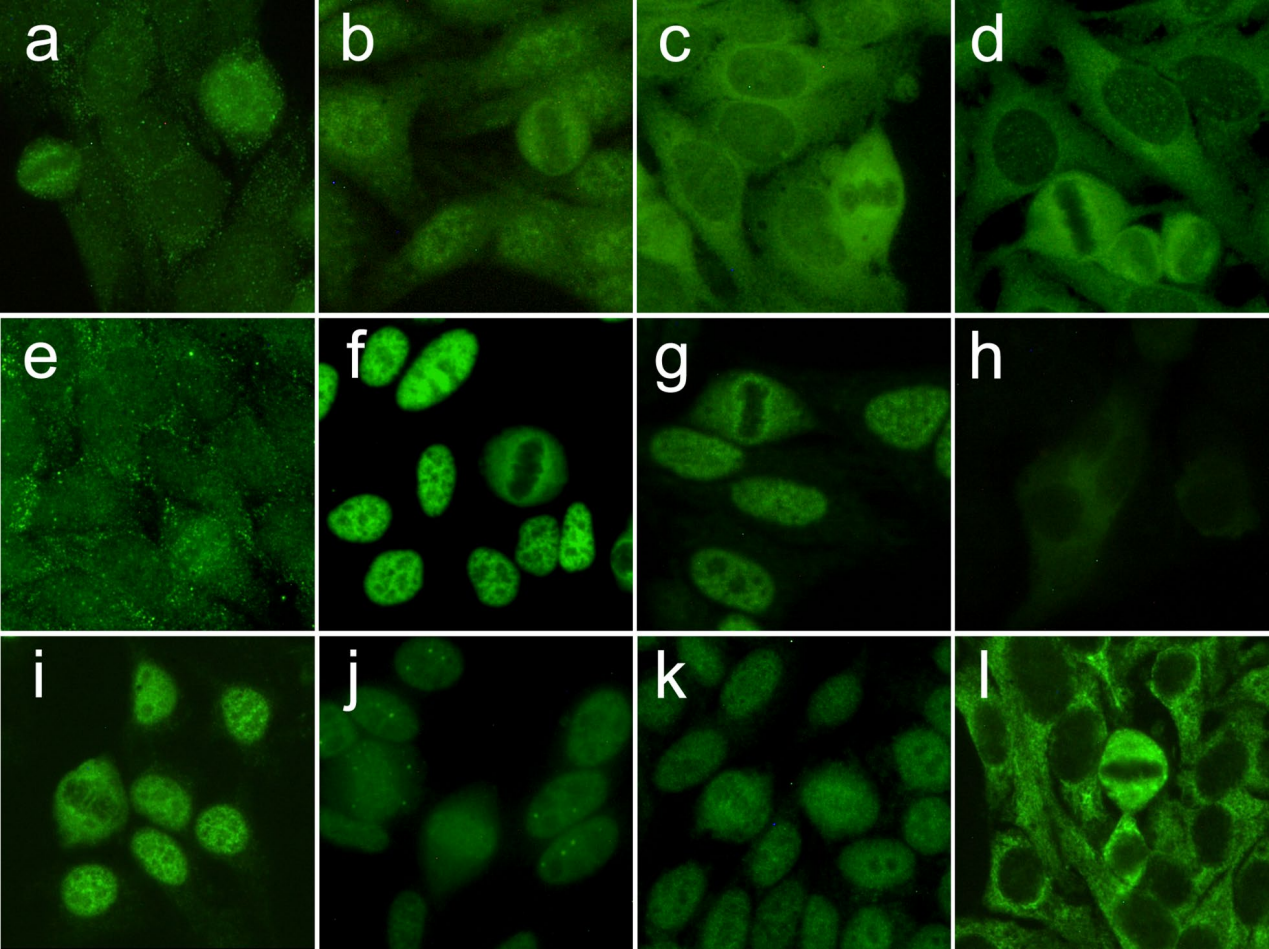
IIF on HEp-2 ANA slides of myositis-specific autoantibodies from patients with IIMs (EUROIMMUN, Lübeck, Germany). **a** Anti-Jo-1 and **b** anti-Jo-1 and anti-Ro52, **c** anti-PL-7, **d** anti-PL-12, **e** anti-KS, **f** anti-Mi-2, **g** anti-SAE-1, **h** anti-MDA5, **i** anti-NXP-2 and **j** anti-NXP-2 with coiled bodies, **k** anti-TIF1γ, **l** anti-SRP

IIF on HEp-2 cells usually demonstrates a cytoplasmic pattern, ranging from fine (Jo-1) to dense fine speckled or homogeneous (PL-7, PL-12) whereas the nucleoplasm is usually negative (Fig. 1a–e) [24]. In these cases, also patients without muscular involvement should be assessed for ARS especially when interstitial lung disease (ILD), arthritis or scleroderma features are present [25].

Anti-Jo-1 is the only autoantibody routinely tested as widely available in most commercial ENA screening assays. An ELISA screening test has been recently developed to identify ARS, with high sensibility and specificity if compared to IP [26] and some commercially available IBs can identify some non-Jo-1 anti-synthetase antibodies [20, 25].

Anti-Jo-1 was first discovered in the ‘80ies in patients with PM [23]. Larger cohorts later demonstrated that its presence was associated with the classical triad of arthritis, myositis and ILD in the majority of patients, in addition to Raynaud’s phenomenon, mechanic’s hands and fever. This clinical presentation together with anti-Jo-1 autoantibodies, led to the description of the antisynthetase syndrome (ASSD), the first attempt to phenotype IIMs in clinical-serologic syndromes [27]. Anti-Jo-1 is detected in 15-25% of patients with polymyositis/dermatomyositis (PM/DM), whereas the other ARS are rarer (anti-PL-7 4-12%, PL-12 < 5%, EJ < 5%, OJ < 5% and only few cases reported with anti-KS, ZO e HA/YRS) [22]. In two-third of the cases, high titers of anti-Ro52 antibodies can be also detected and have been associated with an higher risk of ILD [28].

Clinically, anti-PL-7 patients more frequently present hypo-myopathic features [29, 30], whereas in anti-PL-12 and anti-KS patients the disease can be limited to the lung [31–33]. In a quarter of anti-Jo-1 patients, a symmetrical polyarthritis mimicking rheumatoid arthritis is the main presenting finding and, in some of them, anti-cyclic citrullinated peptide antibodies and rheumatoid factors can be also detected [34].

Regardless of clinical characteristics at presentation (arthritis, myositis and ILD) every patient tends to develop the other features of ASSD when not properly treated [34]. Of note, the lower esophageal sphincter is involved more frequently if compared to the other IIMs [35]. ASSD is histopathologically classified within perimysial immune-myopathies in which perimysium fragmentation and muscle fiber necrosis are the main feature, differently from other DM biopsically characterized by atrophy and vasculopathy [36]. Type I interferon-signature in ASSD is responsible for MHC class I upregulation [37] and MHC class II perifascicular expression [38].

### Anti-Mi-2

Anti-Mi-2 antibodies were the first autoantibodies specific for DM recognized by DID using calf thymus extract [39]. Mi-2 is a helicase of the Nucleosome Remodeling Deacetylase (NuRD) multi-protein complex with nucleosome remodeling and histone deacetylase/demethylase activities [40]. Anti-Mi-2 autoantibodies immunoprecipitate a major protein of 240 kDa, composed by two proteins, Mi-2α and Mi-2β of 220 and 218 kDa, respectively. Other NuRD complex proteins co-precipitate at 200, 150, 75, 65, 63, 50 and 34 kDa [41, 42]. IIF on HEp-2 cells reveals a characteristic fine speckled ANA pattern; during metaphase, chromatin mass is not stained but the nucleoplasm presents the same fine tiny speckles (Fig. 1f). Commercial ELISA and immunoblot kits identify anti-Mi-2 autoantibodies. Anti-Mi-2 are commonly detected in DM patients, either in adults (11–59%) or in children (4–10%), with a great variability among the studies. Their presence in PM and sporadic inclusion body myositis (sIBM) is rarer [40].

The Mi-2 protein is over-regulated during muscle regeneration in DM patients and thought to be related to UV rays exposition, sex and HLA (DRB1*0302 and DRB1*0701) [43–45]. Anti-Mi-2 positive DM patients usually exhibit mild myopathy despite high creatine kinase (CK) levels, without lung involvement and/or cancer [43]. Overall, anti-Mi2 positive is associated with a positive prognosis and a good response to corticosteroids [43].

### Anti-SAE

Small Ubiquitin-like Modifiers (SUMOs) have a key role in post-transcriptional modification of specific proteins in a ubiquitin-like fashion. This process is controlled by the SUMO-Activating Enzyme (SAE), a heterodimer composed of two subunits, SAE-1 and SAE-2 [46], representing the targets of anti-SAE autoantibodies. IP characteristically shows two bands of 40 and 90 kDa, respectively [46, 47]. The IIF ANA pattern is coarse or fine speckled and nucleoli are typically not stained (Fig. 1g) [47].

Anti-SAE are associated with a typical DM phenotype with different prevalence in European (4-10%) and Asian (1–3%) cohorts [48–50], probably due to the strict association with HLADRB1*04-DQA1*03-DQB1*03 haplotypes [51].

The cutaneous involvement is usually severe and typically precedes the muscular involvement. Other clinical relationships cannot be excluded because of the few described cases. However, ILD seems to be rare, whereas severe dysphagia and systemic symptoms have been reported [47]. Only one case series claimed an association with cancer [52].

### Anti-MDA5

Melanoma Differentiation-Associated gene 5 (MDA5) or Interferon-induced helicase C domain-containing protein 1 (IFIH1), is an innate cytosolic sensor, member of the retinoic acid-inducible gene I (RIG-I)-like receptors family (RLRs). MDA5 is able to recognize double-stranded RNA and to initiate signaling events leading to type I interferons production [53].

Anti-MDA5 autoantibodies were firstly detected in IP as a 140 kDa band in a Japanese case series of patients with clinically amyopathic dermatomyositis (CADM) and rapidly progressive interstitial lung disease (RP-ILD). For this reason, they were initially called anti-CADM-140 autoantibodies [54]. Nowadays, ELISA and IB tests are commercially available.

IIF on HEp-2 cells is usually negative. In our experience, a faint fine speckled cytoplasmic fluorescence may be detected in scattered cells (unpublished data) (Fig. 1h).

Clinically, DM anti-MDA5 positive patients present low grade/absent muscle inflammation and acute or subacute RP-ILD [55, 56], which is considered the major negative prognostic factor of this subgroup [57].

MDA5 represents the most frequent target antigen in DM patients of Asian ancestry (10–48% of cases) [58] whereas its prevalence in Europe and USA ranges from 0 to 13%, with great variability among the studies [59–61] and a different clinical presentation. A forthcoming European case series is going to be presented at the European League Against Rheumatism 2018 Congress by Cavagna et al. (unpublished data). A seasonal pattern of CADM has been proposed by Muro et al. [62], suggesting the influence of environmental factors and HLA-DRB1*04:01 and *12:02 have been proposed as further predisposing factors [63].

In addition to classic DM-related cutaneous manifestations, skin involvement is usually severe and characterized by the so called “inverse Gottron papules”, which are tender palmar papules that tend to evolve towards ulcerated-necrotic lesions, with or without digital pulp ulcers [64, 65]. In addition, polyarthritis, recurrent oral aphtosis and diffuse alopecia have been described [66]. A juvenile DM with anti-MDA5 autoantibodies has been also described [67]. No association with malignancies has been demonstrated so far. Macrophage activation syndrome have been described in CADM associated RP-ILD patients. Particularly, a ferritin level of above 1500 ng/mL has been claimed as a predictor of death [68, 69]. Anti-MDA5 autoantibodies titer seems to correlate with disease activity and response to therapy [69].

### Anti-TIF-1

The transcription intermediary factors-1 (TIF-1) family belongs to the tripartite motif-containing proteins (TRIM) superfamily and is involved in multiple biological processes, such as cycle regulation, mitosis and innate immunity [70].

Targoff et al. and Kaji et al. [71, 72] independently described two antibodies directed against a 155 and 140 kDa, rapidly identified as TIF-1γ (TRIM33) and TIF-1α (TRIM24), respectively. Subsequently, a third 120 kDa band, partially overlapping with anti-PL-12, was identified as TIF1β (TRIM28) [73].

IIF on HEp-2 cells demonstrates a fine speckled nuclear pattern (Fig. 1i). ELISA and IB, compared to IP, are reliable test for the detection of anti-TIF-1γ autoantibodies [74]. Two-thirds of the patients present anti-TIF-1γ and anti-TIF-1α autoantibodies, whereas the remaining one-third is positive for anti-TIF-1γ autoantibodies exclusively [70]. Albeit MSAs are claimed to be mutually exclusive, double-positive patients for anti-TIF-1α/Mi-2 autoantibodies have been described [75].

Hyper-expression of TIF-1γ has been found in tumors [76] and regenerating myofibres of DM patients [77]. A meta-analysis demonstrated that anti-TIF-1γ has a 78% sensitivity and 89% specificity for the diagnosis of cancer-associated myositis, with a 58% positive and 95% negative predictive value [78]. The risk of malignancy is higher in patients with anti-TIF-1γ/α than in those with anti-TIF-1γ alone [70].

Clinically, anti-TIF-1 positive patients can be classified in two age groups: (1) younger than 40-year-old patients, with a classical DM at presentation and (2) older than 40-year-old patients, with cancer-associated myositis [70]. Solid tumors, like ovary, lung and breast cancer are the most commonly associated neoplasia, but hematologic disorders and malignancies have been described as well [79]. In general anti-TIF-1γ patients exhibit a hypo-myopathic DM with reduced prevalence of systemic involvement, namely ILD, Raynaud’s phenomenon and arthritis [80]. Conversely, nutcracker esophagus is three times more frequent in anti-TIF-1γ patients than other IIMs [35]. Widespread cutaneous involvement is associated with unique features, such as palmar hyper-keratotic papules, psoriatic-like dermatitis and atrophic hypo-pigmented patches with telangiectasias [80]. An ovoid palatal patch may be present in about one half of patients, more frequently females with cancer-associated amyopathic disease [81].

### Anti-NXP-2

Nuclear matrix protein 2 (NXP-2), encoded by the microrchidia 3 gene, is a 140 kDa protein involved in epigenetic regulation, RNA metabolism and preservation of nuclear chromatin architecture [82]. Anti-NXP-2 autoantibodies were found in a cohort of juvenile DM patients as a 140 kDa protein firstly named anti-MJ [83].

IIF on HEp-2 cells reveals a fine speckled nuclear pattern (Fig. 1k). Moreover, a nuclear dots pattern is detectable in 60% of sera [84], due to co-localization of NXP-2 with pro-myelocitic leukemia (PML) bodies [85] (Fig. 1j).

Anti-NXP-2 antibodies have been initially associated with a severe juvenile DM complicated by calcinosis, polyarthritis and intestinal vasculitis [86]. More recently, they have been also found in adult patients, with variable prevalence from 1.6 to 17% [87–89]. Anti-NXP-2 autoantibodies show a bimodal spectrum of clinical association, with calcinosis being more frequent in younger patients and cancer more common in the elderly [90], especially in male gender [88], even though with a lower prevalence when compared to anti-TIF-1 [87, 88].

### Anti-SRP

The signal recognition particle (SRP) is a complex of six proteins (9, 14, 19, 54, 68 and 72 kDa) and a 300 nucleotides long RNA (7SL RNA) involved in the recognition and transportation of proteins to the endoplasmic reticulum [91]. Anti-SRP autoantibodies are more frequently directed against the SRP-54 fragment, albeit anti-SRP-68, anti-SRP-72 and anti-7SL RNA autoantibodies have been also described [91].

**Fig. 2:**
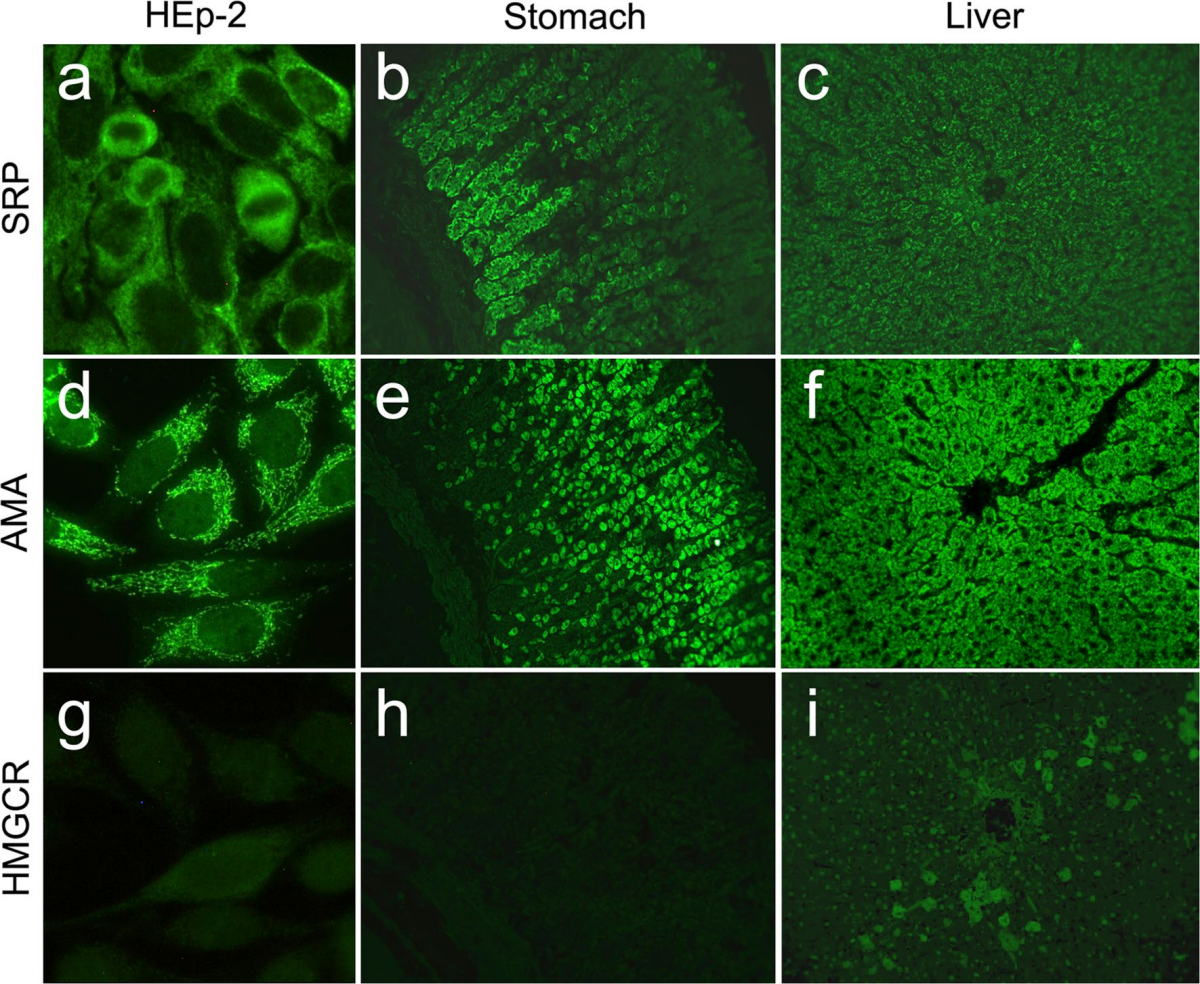
Immunofluorescence patterns from patients with IMNMs with anti-SRP and anti-HMGCR antibodies on HEp-2 ANA slides (EUROIMMUN, Lübeck, Germany) and rat liver and stomach slides (DiaSorin, Italy), compared to AMA-M2 antibodies. **a** Anti-SRP cytoplasmic dense fine speckled pattern on HEp-2, **b** chief cells on stomach and **c** fine granular liver staining; **d** AMA-M2 cytoplasmic reticular on HEp-2, **e** granular staining of parietal stomach cells and **f** diffused fluorescence of hepatocytes; **g** Anti-HMGCR faint cytoplasmic fluorescence on few numbers of HEp-2 cells, **h** negative stomach and **i** fine cytoplasmic fluorescence of scattered hepatocytes around the biliary ducts

A dense fine speckled cytoplasmic pattern has been associated with the presence of anti-SRP (Fig. 2a); moreover, IIF on stomach–liver–kidney rat sections demonstrates a cytoplasmic staining of gastric chief cells (Fig. 2b) and hepatocytes (Fig. 2c) [92].

Anti-SRP-54 autoantibodies ELISA tests are commercially available, but they are less sensitive than IP [93]. Anti-SRP antibodies can also be tested on LIA assays, however careful temperature control is necessary in order to avoid false positive results [19].

These autoantibodies are strongly associated with immune-mediated necrotizing myositis (IMNM) where they may play a pathogenic role [94]. Histopathologically, they are characterized by scarce inflammatory CD8+ endomysial infiltrate, class I MHC upregulation, necrosis and myofiber regeneration [36] with poor response to therapy, mimicking muscular dystrophy [95]. Being strongly associated to HLA-DR5, the prevalence of anti-SRP autoantibodies is higher in Asian (8–13%) than in European patients [96]. Prevalence of anti-SRP in IMNM is highly variable among studies, ranging from 0 to 54%, because of differences regarding the type of assay (IB vs IP), low number of patients and genetic background [97–100]. In addition, esophageal involvement is common [101], whereas the possibility of a prominent cardiac involvement has not been yet confirmed [99, 102]. Lung involvement has been reported in a few cases [103], as well as an overlap with anti-synthetase antibodies like PL-12 and anti-Jo-1 [104, 105]. Intriguingly, autoantibodies level correlates to disease activity, CK levels and response to therapy [106].

### Anti-HMGCR

The 3-hydroxy-3-methylglutaryl-coenzyme A reductase (HMGCR) is the rate-controlling enzyme of the mevalonate pathway, bringing to the production of cholesterol. Of note, HMGCR is the same enzyme targeted by statins. Autoantibodies towards a complex 200/100 kDa band were first described in patients with IMNM, and only after identified as anti-HMGCR autoantibodies [107].

IIF pattern is difficult to recognize. In a minority of cases finely granular cytoplasmic staining with a perinuclear reinforcement is visible on a small number of scattered cells (3% of the total cellularity) (Fig. 2g). On rat liver, a scattered cytoplasmic staining of hepatocytes around the liver lobules, namely anti-HMGCR Antibody Associated Liver Immunofluorescence Pattern (HALIP), can be noted (Fig. 2i) [108, 109]. Anti-HMGCR antibodies can be identified with different immunoenzymatic technologies, such as ELISA, CLIA, IB or ALBIA [110].

A history of statin exposure is not mandatory to develop anti-HMGCR positive IMNM, being of some relevance only in patients older than 50 [111, 112]. Anti-HMGCR antibodies are not found in self-limiting statin associated myopathy [113], albeit they may be associated with an increased risk of cancer [114]. An association with the DRB1*11:01 haplotype has been demonstrated, whereas DQA1 and DQB1 seem to have a protective role [115].

Anti-HMGCR positive patients present with a typical IMNM, responds well to immunosuppressive therapy and intravenous immunoglobulins [116], but tend to relapse after tapering [111]. Younger patients experience more severe disease with worse prognosis [117]. Autoantibody titers seem to correlate with CK levels, muscular weakness and response to therapy [111].

## Myositis-associated autoantibodies

Numerous MAAs have been described so far. Characteristically, they can be found in IIMs, albeit not specific as found in other CTDs [118].

### Anti-PM-Scl

Anti-PM-Scl autoantibodies are directed against the exosome, a macromolecular nucleolar complex composed by 11–16 proteins (from 20 to 110 kDa) that degrades mRNA. The two pivotal proteins of the complex are PM-Scl-75 and PM-Scl-100. IP represents the gold standard for their determination. Historically, an ID test after positive nucleolar staining in IIF was used to confirm anti-PM-Scl reactivity.

**Fig. 3:**
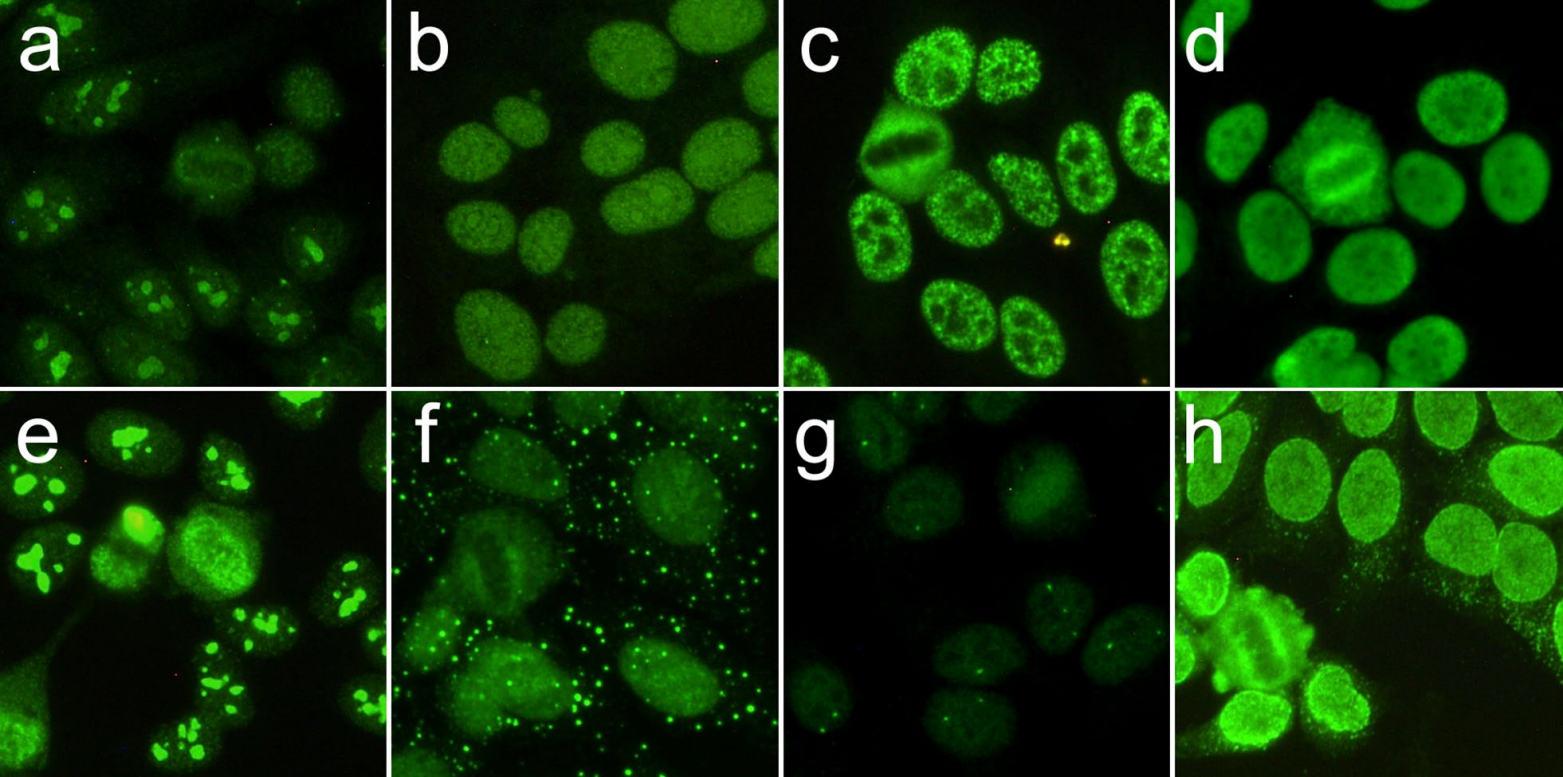
Myositis-associated autoantibodies and some peculiar IIF pattern on HEp-2 ANA slides (EUROIMMUN, Lübeck, Germany). **a** anti-PM-Scl-100, **b** anti-PM-Scl-75, **c** anti-U1-snRNP, **d** anti-Ku, **e** anti-fibrillarin, **f** cytoplasmic discrete dots as seen in anti-Su/Ago2, **g** nuclear few nuclear dots as seen in anti-SMN, **h** punctate nuclear envelope as seen in anti-NUP

PM-Scl-100 and PM-Scl-75 were identified in 1992 and, in the following years, the immuno-dominant epitope PM1α was cloned and employed to develop reliable and specific ELISA tests [119]. PM1α ELISA and PM-Scl-100 LIA tests show concordance with IP at high level (> 90 and 98.3%, respectively), whereas PM-Scl-75 LIA has a lower specificity, especially when considering PM/SSc overlap syndromes [120]. Single positivity against PM-Scl-75 or -100 can be detected and associates with different disease phenotypes. HEp-2 IIF typically shows a mixed homogeneous nucleolar and fine speckled nuclear pattern when anti-PM-Scl-100 are present, whilst anti-PM1-α and PM-Scl-75 may show both nucleolar and non-nucleolar patterns (Fig. 3a, b).

PM-Scl autoantibodies are found in 4–12% of adult patients with myositis [121, 122] with low prevalence in Asiatic and paediatric cohorts [123]. Their presence has been associated with HLA-DQA1*0501, DQB1*02 and DRB1*0301 alleles [122].

Despite their presence in many connective tissue diseases, these autoantibodies are typically present in PM/SSc overlap syndromes with an increased risk of Raynaud’s phenomenon, arthritis, mechanic’s hands and ILD [124]. In detail, isolated anti-PM-Scl-75 have been more frequently found in patients with joint contractures and SSc, higher CK levels associate with anti-PM-Scl-100, whereas the simultaneous presence of anti-PM-Scl-75 and -100 are linked to muscle involvement, digital ulcers and ILD but lower prevalence of lung hypertension [125].

Autoantibodies directed against C1D, an exosome associated protein, were detected by ELISA and Western blot analysis in 23% of a PM/SSc overlap syndrome cohort, with frequencies comparable to anti-PM/Scl antibodies [126].

### Anti-RNP

The RNP/Sm complex comprises several proteins (70 kD, A, A′, B, B′, B″, C, D, E, F, G) and five RNA (U1, U2, U4, U5 and U6). U1 RNA interacts with 70 kD, A and C to create the U1-snRNP [127]. High-titre anti-U1-snRNP and in particular when targeting the 70kD protein are considered specific markers of mixed connective tissue disease (MTCD), whereas low titres can be found in other CTDs [127].

Many home-made or commercial assays can detect anti-RNP autoantibodies, with differences among immunoassays depending on the immobilized antigen [127]. Usually, anti-U1-snRNP (more often the 70k subunit) and the anti-Sm (typically the D subunit) are the only autoantibodies tested in clinical practice. Large speckled and large coarse speckled are the most frequent HEp-2 IIF patterns observed (Fig. 3c).

Patients with myositis may exhibit anti-U1-snRNP positivity, especially those with a mild disease [128, 129]. They are usually steroid-responsive, even though ILD and/or neurological involvement may be part of the clinical presentation [128, 129]. Whether the only presence of anti-U1-snRNP and myositis has to be considered an incomplete form of MTCD or a true myositis, is still a matter of debate [128, 129]. In addition, anti-U2-RNP [130], U5-RNP [131] and anti-U4/U6-RNP [132] have been described in patients with PM/SSc overlap syndrome.

### Anti-fibrillarin

Fibrillarin, a highly conserved nucleolar 34 kDa protein involved in the processing of ribosomal RNA, is part of the U3-small nucleolar (sno)-RNP complex together with other proteins and U3 RNA. Fibrillarin is the primary target of anti-U3-snoRNP autoantibodies [133]. IP is the gold standard for its detection showing good concordance with IB assays that use the recombinant protein [134].

HEp-2 IIF demonstrates a typical “clumpy” nucleolar pattern, with jagged staining of the nucleoli, coiled bodies and peri-chromosomal staining at the metaphase plates [135] (Fig. 3e).

Anti-fibrillarin antibodies are detected in a small percentage of SSc patients and rarely in SLE, primary Raynaud’s phenomenon and myositis [136]. In detail, they identify a subset of SSc patients more often of African origin, with serious cutaneous and visceral involvement and a higher prevalence of myositis [137, 138].

### Anti-Ku

The Ku protein, involved in the canonical non-homologous end-joining pathway of the DNA repair, is a heterodimer consisting of the two subunits, 70 and 80 kDa [139]. Anti-Ku can be identified with numerous assays, such as ELISA, CIE or IB. IIF demonstrates a fine speckled nuclear pattern with a peculiar ring beam surrounding the metaphase on HEp-2 cells and a clumpy speckled pattern on primate’s liver [140] (Fig. 3d).

Anti-Ku autoantibodies have been identified in 9-19% of the patients with PM/SSc overlap syndromes and SLE, being associated with arthralgia, Raynaud’s phenomenon and ILD [141, 142]. Of note, whilst muscular involvement seems to be steroid-sensitive, ILD is more frequently progressive, severe and steroid-resistant [143].

### Anti-Ro

Antibodies directed against the ribonucleoproteic complex SSA/Ro and SSB/La have been originally identified in SjS and SLE. Actually, antigen Ro is made by two separate complexes of 52 and 60 kDa called Ro52/TRIM21 and SSA/Ro60, respectively. Antigen SSB/La has a molecular weight of 48 kDa [144].

ANA may result falsely negative on traditional HEp-2 cells when isolate anti-Ro are present, because Ro52 is a cytoplasmic antigen and Ro60 may be lost during the preparation. For this reason, human SSA/Ro60-transfected HEp-2 cells (HEp-2000) are sometimes used [145, 146]. Otherwise, a characteristic pattern defined as “myriad discrete fine speckled” may be observed [147]. Anti-SSB autoantibodies show a similar pattern [148].

Anti-Ro52 can be found in IIMs [149] and are frequently associated with other MSAs, in particular anti-synthetase [28], anti-MDA5 [61] and anti-SRP autoantibodies [12].

They are known to be a negative prognostic factor regarding systemic involvement such as ILD, whereas their role in the severity of muscular involvement has not been identified [13]. Anti-Ro52 autoantibodies are known to be associated with atrioventricular congenital heart block [150].

### Anti-cN-1A

Cytosolic 5′nucleotidase 1A (cN-1A o NT5C1A) is a protein involved in the hydrolysis of adenosine monophosphate, controlling energy and metabolic cell balance [151]. Anti-cN-1A autoantibodies, first called anti-Mup44, were simultaneously described by Salajegheh et al. and Pluk et al. [152, 153] as targeting a 44 kDa protein in patients with sIBM.

These autoantibodies were initially detected by immunoblotting from purified skeletal muscle extracts [153]. A novel standardized IgG ELISA is now available [154]. In addition to IgG, circulating IgA and IgM anti-cN-1A autoantibodies have been recognized [155]. IIF ANA pattern is still undefined.

Anti-cN-1A autoantibodies are demonstrated in one-third of the patients with sIBM and in less than 5% with other IIMs or neuromuscular diseases [151]. A recent study demonstrated that positive anti-cN-1A sIBM patients are included in a more severe sIBM subtype and represent a homogeneous group as exhibiting higher mortality risk, less proximal upper limb weakness (not typical of sIBMs) and a cytochrome oxidase deficiency in muscular fibers, when compared to negative patients [156].

It is not known whether they have to be considered as MSAs or MAAs as also demonstrated in other autoimmune diseases, such as SjS (30%) and SLE (20%) [157]. Furthermore, they have been recently demonstrated in a cohort of severe juvenile myositis with lung involvement, juvenile idiopathic arthritis, but also in 12% of healthy children [158].

Despite low sensitivity, anti-cN-1A autoantibodies are high specific and highly predictive of sIBM [159] thus being of particular importance when bioptic specimens are not diagnostic.

## Miscellaneous autoantibodies in IIM

Several other autoantibodies have been identified as associated with IIMs, but little is known about their clinical relevance. In fact, they are not routinely determined because easy-to-perform routine specific immunoassays still lack and they are rarely found.

### Anti-RuvBL1/2

RuvBL1 (49kD) and RuvBL2 (48kD) constitute a nuclear complex involved into DNA repair and transcription. Two distinct bands of approximately 50 kDa are found in IP [160]. By means of ELISA and/or IB techniques, anti-RuvBL1/2 have been found in several CTDs, but those involved in SSc and myositis recognize different conformational epitopes identified by IP exclusively [160]. On HEp-2 cells, a fine speckled pattern is associated with these antibodies, with increased fluorescence in prophase and decreased in metaphase. Additionally, a fine speckled pattern can be found in the cytoplasm of about 40% positive sera [160]. Anti-RuvBL1/2 antibodies are highly specific for SSc, are associated with PM/SSc overlaps with diffuse cutaneous sclerosis and more frequently found in older patients of male sex [160–162] or, less frequently, in necrotizing polymyositis with morphea [162].

### Anti-Su/Ago2

Anti-Su/Argonaute-2 (anti-Su/Ago2) autoantibodies have been originally identified in SLE patients by means of immunodiffusion technique in the late ‘80ies [163]. Although their high prevalence in CTDs, few studies are available.

By IP, two distinct 100 and 102 kDa adjacent bands can be seen in addition to a further 200 kDa band [164]. Argonaute-2 protein constitutes the 100 kDa band and plays a key role in miRNA and interference RNA maturation and metabolism [163]. Argonaute-2 colocalized with GW bodies, a cytoplasmic organelle associated with RNA metabolism [164]. Its location and function is responsible for the particular cytoplasmic pattern of these autoantibodies also known as “GW-bodies-like” or “cytoplasmic discrete dots” (Fig. 3f) [164].

Anti-Su/Ago2 autoantibodies are frequently associated with other MSA or MAA antibodies, in particular ARS, anti-TIF-1γ and anti-MDA5 [165]; anti-Ro52 antibodies are found in almost one half of the patients [166]. It has been reported that anti-Su/Ago2 antibodies can be demonstrated in about 7.5% of the patients of Japanese origin. Apparently, there is no statistical difference between anti-Su/Ago2 positive and negative patients; however, a correlation seems to exist with ILD and absence of cancers [165].

### Anti-SMN

The Survival of Motor Neuron (SMN) is a multi-ribonucleoproteic complex able to interact with the RNP-complex related D–E–F-G proteins. The SMN complex is involved into the assembly of snRNPs and co-localizes with Cajal bodies. These autoantibodies have been first described in a small number of PM patients negative for anti-U1-snRNP and/or anti-Sm but positive for RNP D–E–F-G bands by IP. This observation was indeed responsible for the identification of other SMN-complex components, namely Gemin 2 (33 kDa), Gemin 3 (130 kDa), Gemin 4 (120 kDa) and SMN itself (38 kDa) [167].

Anti-SMN antibodies typically exhibit a few nuclear dots pattern on HEp-2 cells with well distinguished 2–7 nuclear dots, similarly to anti-p80-coilin, anti-NXP2 and anti-PML pattern, seldom associated to cytoplasmic or nuclear speckled patterns (Fig. 3g).

It is not clear whether positive patients exhibit distinct clinical features. Anyhow, in the original small group of positive patients [167] and a small Italian cohort [20], a PM/SSc overlap syndrome was present. It is of note that SMN-complex genetic mutations are frequently found in neuromuscular degenerative diseases such as spinal-muscle atrophy. That is why anti-SMN autoantibodies are of relevance in basic research [168].

### Anti-NPC

Nuclear pore complex (NPC) regulates protein and RNA trafficking to the nucleus. It is constituted by a complex of several proteins including Nup358/RanBP2, Nup90, Nup62 and gp210 [169]. Anti-NPC autoantibodies, and, in particular, anti gp210 are typically associated to Primary Biliary Cholangitis (PBC) and Autoimmune Hepatitis (AIH) [170]. In a cohort study from Canada, anti-NPC antibodies were found in a PM/SSc overlap syndrome and called anti-NUP Syndrome, which was found to be associated with HLA-DQ1*0501. In this case, a typical nuclear speckled laminar pattern on HEp-2 cells was observed [171] (Fig. 3h).

### AMA-M2

Among the ten different anti-mitochondrial antibodies (AMA), called M1–M10, anti-M2 antibodies (AMA-M2) are the hallmark of PBC [172]. However, they can be also found in 7–12% of IIM patients without PBC [173]. AMA antibodies are readily detectable on HEp-2 cells as they display a pathognomonic cytoplasmic reticular pattern, and in triple tissue slides (Fig. 2d–f). In a Japanese study, the presence of AMA-M2 in the course of IIM was associated with muscle atrophy, granuloma formation [173] and heart involvement with high risk of supraventricular arrhythmias [174]. A distinct inflammatory phenotype associated with chronic skeletal muscle disease and severe cardiac involvement was also found in a North American cohort [175]. These associations have not been confirmed in an European series [176].

### Other antibodies

Several cytoplasmic autoantibodies are described in IIM patients such as anti-KJ towards a 30/43 kDa translocation factor [177], anti-Fer directed against the elongation factor 1 and anti-Wa recognizing a 48 kDa cytoplasmic protein with still unknown function [132]. All these antibodies are typically found in anti-synthetase-like syndromes. Anti-Mas antibodies are directed against a selenocysteine-containing tRNA complex lacking any tRNA-synthetase activity but involved in protein translocation. The band of precipitation is found at 48 kDa [178]. These antibodies have been described in AIH and in a single patient with non-immune mediated rhabdomyolysis [178].

DNA-repairing complexes, especially mismatch-repair complexes such as PMS1, PMS2 and MLH1, are frequently recognized as target antigens in IIMs [179]. Initially defined as MSAs, they do indeed frequently associate with other MSAs, in particular anti-Mi-2, but they can also be found in other non-muscular diseases, such as SLE. They generally mark mild disease [180].

Anti-cortactin antibodies have been initially found in IIM patients characterized by the simultaneous presence of anti-MDA5 or anti-HMGCR antibodies by ELISA [181]. As blot confirming assay identified an unexpected 68 kDa band, it was then found that MDA5 and HMGCR extracts used in the ELISA tests were contaminated by cortactin [181]. Anti-cortactin antibodies were originally found in myasthenia gravis [182] and later in IIM patients (about 20%) and other systemic connective tissue diseases [181].

Anti-Four-and-a-Half LIM domain 1 (FHL1) antibodies were identified in about 25% of IIM patients. These antibodies associated with a severe prognosis, muscle atrophy, vasculitis, dysphagia and advanced muscular damage. Curiously, FHL1 mutations cause hereditary X-linked congenital myopathies [183].

## Conclusions

Although autoantibodies are considered to be epiphenomenon in autoimmunity, their presence frequently plays a pivotal role for the diagnosis of these diseases. Indeed, several of them exhibit a pathogenethic role in IIMs. Despite this, there is still a gap between bench and bedside because the intense basic research efforts have not been translated in clinical practice, as already futuristically underlined more than 20 years ago [96, 184]. As a fact, only anti-Jo-1 have been included into the 2017 Classification Criteria for Adult and Juvenile IIMs [3].

Remarkably, in the context of heterogeneously grouped diseases such as myositis, they should be even more appreciated as able to clinically stratify patients in terms of diagnostic work-up, histological patterns, peculiar organ involvement, severity, and, therefore, treatment intensity and prognosis. This process could be accomplished by a laboratory auto-immunologist [185] well-trained in recognition of IIF ANA nuclear and, also, cytoplasmic patterns, in strict collaboration with the clinical doctor, as a decision-maker for running in-depth analysis towards the identification of the culprit autoantibody.

In addition, multicentric studies with a multidisciplinary approach may help bridging the divide of the selection bias depending on the setting where patients are initially screened (i.e. pneumologic vs. dermatologic vs. immuno-rheumatologic vs. neurologic outpatient clinics).

## Abbreviations

SLE: systemic lupus erythematosus
CTD: connective tissue disease
SjS: Sjögren syndrome
SSc: systemic sclerosis
IIM: idiopathic inflammatory myopathies
PM: polymyositis
DM: dermatomyositis
MSA: myositis-specific autoantibodies
MAA: myositis-associated autoantibodies
ARS: anti-synthetase autoantibodies
IPAF: interstitial pneumonia with autoimmune features
IIF: indirect immuno-fluorescence
CIE: counter-immuno-electrophoresis
ID: immuno-diffusion
ELISA: enzyme-linked immunosorbent assay
FEIA: fluorescent enzyme-linked assay
CLIA: chemiluminescent immuno-assay
IP: immuno-precipitation
ALBIA: addressable laser beads immunoassay
ANA: anti-nuclear autoantibodies
IB: immunoblot
LIA: line blot assays
tRNA: transfer RNA
DID: double immune-diffusion
ILD: interstitial lung disease
ASSD: antisynthetase syndrome
PM/DM: polymyositis/dermatomyositis
NuRD: nucleosome remodelling deacetylase
sIBM: sporadic inclusion body myositis
CK: creatine kinase
SUMOs: small ubiquitin-like modifiers
SAE: SUMO-activating enzyme
MDA5: Melanoma Differentiation-Associated gene 5
RIG-I: retinoic acid-inducible gene I
RLR: RIG-I-like receptors family
RP-ILD: rapidly progressive interstitial lung disease
CADM: clinically amyopathic dermatomyositis
TIF1: transcription intermediary factors-1
TRIM: tripartite motif-containing proteins
NXP-2: nuclear matrix protein 2
PML: pro-myelocitic leukaemia bodies
SRP: signal recognition particle
IMNM: immune-mediated necrotizing myositis
HMGCR: 3-hydroxy-3-methylglutaryl-coenzime A reductase
HALIP: anti-HMGCR antibody associated liver immunofluorescence pattern
MTCD: mixed connective tissue disease
sno: small nucleolar
cN-1A: cytosolic 5′nucleotidase
SMN: survival of motor neuron
NPC: nuclear pore complex
PBC: primary biliary cholangitis
AIH: autoimmune hepatitis
FHL1: anti-four-and-a-half LIM domain 1
AMA: anti-mitochondrial antibodies
